# The Endophyte *Trichoderma asperellum* M2RT4 Induces the Systemic Release of Methyl Salicylate and (*Z*)-jasmone in Tomato Plant Affecting Host Location and Herbivory of *Tuta absoluta*

**DOI:** 10.3389/fpls.2022.860309

**Published:** 2022-04-05

**Authors:** Ayaovi Agbessenou, Komivi S. Akutse, Abdullahi A. Yusuf, Fathiya M. Khamis

**Affiliations:** ^1^International Centre of Insect Physiology and Ecology, Nairobi, Kenya; ^2^Department of Zoology and Entomology, University of Pretoria, Hatfield, South Africa; ^3^Forestry and Agricultural Biotechnology Institute, University of Pretoria, Hatfield, South Africa

**Keywords:** *in planta* colonization, plant-insect-microbe interactions, constitutive and induced defenses, semiochemicals, behavior, biological control

## Abstract

The use of endophytic fungi has dramatically increased plant performance through the enhancement of plant protection against abiotic and biotic stressors. We previously demonstrated that the endophytic fungus *Trichoderma asperellum* M2RT4 improves tomato defenses against the tomato leafminer *Tuta absoluta* through the reduction of oviposition, leafmining, pupation, and adult emergence. However, the underlying mechanism by which the presence of this endophytic fungus within tomato host plant affects *T. absoluta* host selection and life-history traits is unknown. We tested the behavioral responses of *T. absoluta* in Y-tube olfactometer bioassays and found that females preferred non-inoculated tomato plants against those inoculated by endophytes. Additionally, *T. absoluta* females were not attracted to non-inoculated infested nor to inoculated-infested tomato plants. Chemical analysis revealed the emission of methyl salicylate in inoculated tomato plant and an increase in the amounts of monoterpenes emitted from non-inoculated infested plants. Additionally, we found that upon herbivory, *T. asperellum* M2RT4 modulates tomato plant chemistry through the production of (*Z*)-jasmone thus activating both salicylic and jasmonic acid defense pathways. Further, *T. absoluta* females were attracted to monoterpernes including α-pinene, 2-carene, and β-phellandrene but repelled by methyl salicylate. Methyl salicylate could therefore be considered as a good semiochemical-based candidate for sustainable *T. absoluta* management using a “push-pull” approach. However, in dose-response bioassays, females of *T. absoluta* did not show any preference to the four component-blend (α-pinene, 2-carene, β-phellandrene, and methyl salicylate). (*Z*)-jasmone-treated tomato leaflets significantly reduced the leafmining activity of the pest at the concentration of 10 ng/μL and causing the highest larval mortality rate (83%) with the shortest LT_50_ (1.73 days) 7 days post-treatment. *T. asperellum* M2RT4 effect on herbivore performance was then (*Z*)-jasmone-mediated. These findings expand our understanding of how the endophytic fungus *T. asperellum* M2RT4 could mediate chemical interactions between *T. absoluta* and its host plant which are potentially important for development of environmentally friendly *T. absoluta* management programs.

## Introduction

Plants represent a rich source of nutrients for insects and are known to emit a cascade of chemical cues which are used by herbivorous insects to locate their host and find suitable oviposition sites (Bruce et al., 2005). As well, plants are closely associated with a diversity of beneficial microorganisms living within their tissues, some of which offer protection against herbivorous insects (Barelli et al., 2016). Upon herbivory, plants respond by releasing defense compounds termed herbivore-induced plant volatiles (HIPVs) which have been reported to often vary quantitatively and qualitatively (Rostás et al., 2006; Shrivastava et al., 2015). Herbivore-induced plant volatiles are explored for use in pest management programs, particularly for improving biological control (Lin et al., 2017; Ayelo et al., 2021a).

The tomato leafminer *Tuta absoluta* (Meyrick) (Lepidoptera: Gelechiidae) which is native to South America, has become an invasive and serious economic pest of tomato and several crops belonging to the Solanaceae family in Africa (Desneux et al., 2010; Idriss et al., 2020). Females of *T. absoluta* primarily rely on host plant volatiles to locate and select suitable oviposition sites (Proffit et al., 2011) where eggs are laid on the upper surface of the leaves. Upon hatching, the larvae feed voraciously on the leaves and fruits of the crop, interfering with photosynthetic activity, nutrient transport, and eventually creates avenues for entry of opportunistic microorganisms (Desneux et al., 2010). It is reported that the tomato leafminer causes up to 100% crop losses estimated at USD 1.1 billion worldwide annually (Pratt et al., 2017). Synthetic chemical insecticides remain the primary control strategy used against *T. absoluta* in Africa, but the growing threat of insecticide resistance coupled with public concerns over non-target and beneficial organisms associated with their indiscriminate use (Guedes et al., 2019) prompted the urgent need to develop alternative, ecofriendly and sustainable control tools (Agbessenou et al., 2020). Antagonists such as endophytic fungi are considered a safer alternative to synthetic insecticides due to their reduced toxicity and lower chances of resistance (La Spada et al., 2020). Endophytic fungi are common, ubiquitous and widespread microorganisms that live within their host plants (Vega, 2008; Poveda, 2021a) and have been reported to provide *ab initio* protection which represents a state of heightened defense throughout the plant (Wu et al., 2017; Wei et al., 2020). It has been shown that the presence of endophytes in host plants often elicit induced systemic resistance probably as a result of the modification of the plant’s physiology and biochemistry which subsequently enhances defense against herbivorous insects (Rostás et al., 2006; Pineda et al., 2013).

*Trichoderma asperellum* is an opportunistic, asymptomatic microbial endophytic fungus that lives within a wide range of host plants (Siddaiah et al., 2017; Poveda, 2021a) which can successfully colonize tomato and nightshade plant tissues (Agbessenou et al., 2020). *Trichoderma* spp. have been reported to increase host plants resistance against below- and above-ground biotic stressors such as the root-knot nematode, *Meloidogyne incognita* and the southern green stink bug, *Nezara viridula* (Hemiptera: Pentatomidae) (Pineda et al., 2010; Affokpon et al., 2011; Alınç et al., 2021; Poveda, 2021b). Additionally, *Trichoderma* species produce secondary metabolites with biological activity against herbivores (Vinale et al., 2012; Poveda, 2021b) and can influence plant defense chemistry, including the constitutive and induced expression of phytohormones such as jasmonic acid (JA), salicylic acid (SA), abscisic acid (ABA), and ethylene (ET) which are known to regulate plant defense response to herbivory (Jallow et al., 2008; Davis et al., 2013; Kottb et al., 2015; Poveda et al., 2020). Among these metabolites, JA and SA play an important role in the regulation of cellular immune responses in plants (Singh et al., 2019). Jasmonic acid belongs to a collective group of cyclopentanone plant hormones (jasmonates) that are known to regulate a variety of processes in plant development and inducing insecticidal activities in plants (Black et al., 2003; Degenhardt et al., 2010). On the other hand, SA regulates defense systems against pathogens and herbivores with piercing-sucking mouthparts, such as aphids (Homoptera: Aphididae) (Loake and Grant, 2007). We previously reported that when present in both tomato and nightshade plants, *T. asperellum* M2RT4 triggered the systemic protection of both host plants against the tomato leafminer *T. absoluta* through the reduction of adult oviposition and leafmining activity (Agbessenou et al., 2020). However, the underlying mechanisms mediating these interactions between endophytic fungi and how they prime plant defense systems especially in the tomato-*T. absoluta* model is not yet elucidated. Therefore, this study aims at identifying volatile compounds (including phytohormones) released by inoculated tomato plant that prime defense responses against attack of both adult and immature stages of *T. absoluta*. We hypothesized that *T. asperellum* inoculated tomato plants trigger the production of phytohormones (JA and SA) which could reduce the attractiveness to *T. absoluta* females while negatively affecting leafmining activity of the pest. To test this hypothesis, we: (i) investigated the behavioral responses of *T. absoluta* females to non-inoculated, inoculated, non-inoculated infested, and inoculated infested tomato plant volatiles, (ii) identified the odor profile associated with non-inoculated and inoculated tomato plants under *T. absoluta* attack, (iii) tested the behavioral responses of *T. absoluta* females to synthetic compounds of the volatile organic compounds (VOCs), and (iv) assessed the ability of (*Z*)-jasmone to protect tomato plant from *T. absoluta* herbivory.

## Materials and Methods

### Fungal Culture

*Trichoderma asperellum* M2RT4 obtained from the International Centre of Insect Physiology and Ecology (*icipe*) Nairobi, Kenya’s Arthropod Pathology Unit Germplasm was cultured on potato dextrose agar (PDA) (OXOID CM0139, Oxoid Ltd., Basingstoke, United Kingdom), and maintained at 25 ± 2°C in complete darkness. Conidia were harvested by scraping the surface of 2–3-week-old sporulated cultures using a sterile spatula. The harvested conidia were then suspended in 10 mL sterile distilled water containing 0.05% Triton X-100 (MERCK KGaA, Darmstadt, Germany) and vortexed for 5 min at about 700 rpm to break conidial clumps and ensure a homogenous suspension (Akutse et al., 2013; Agbessenou et al., 2020). Conidial concentrations were quantified using an improved Neubauer hemocytometer under a light microscope (Goettel and Inglis, 1997). The conidial suspension was adjusted to a concentration of 1 × 10^8^ conidia mL^–1^ through serial dilution prior to inoculating the tomato seeds.

Prior to commencement of the bioassays, spore viability was determined by plating 0.1 mL of 3 × 10^6^ conidia mL^–1^ evenly onto a 9-cm Petri dishes containing PDA. Three sterile microscope cover slips (2 cm × 2 cm) were placed on the surface of the inoculated plates. Plates were then sealed with Parafilm and incubated in complete darkness at 25 ± 2°C and were examined after 16–20 h. Germination rate (%) of conidia was determined from 100 conidia that were randomly selected on the surface area covered by each cover slip under a light microscope (×400) using the method described by Goettel and Inglis (1997). Conidia were considered to have germinated when the length of the germ tube was at least twice the diameter of the conidium. The experiment was replicated four times.

### Inoculation of Seeds and Assessment of Colonization by the Endophyte *Trichoderma asperellum* M2RT4

The tomato *Solanum lycopersicum* cultivar Moneymaker, used in the present study was purchased locally (Simlaw Seeds Company Ltd., Nairobi, Kenya). Seeds were surface-sterilized by washing them successively in 70% ethanol for two minutes followed by 1.5% sodium hypochlorite for another three minutes and finally rinsed three times in sterile distilled water. Thereafter the seeds were placed on sterile filter paper in a cabinet until the residual water evaporated. Effectiveness of the surface sterilization technique was confirmed by plating out 0.1 mL of the last rinse water onto potato dextrose agar and also imprinting of surface sterilized seeds onto PDA (tissue imprint) supplemented with 100 mgL^–1^ Streptomycin and plates were incubated at 25°C for 14 days (Schulz et al., 1998). Seeds were then soaked overnight for 12 h in conidial suspensions titrated at 1 × 10^8^ conidia mL^–1^. Sterilized seeds soaked overnight for 12 h in sterile distilled titrated (0.05% Triton X-100) water were used as the control (Agbessenou et al., 2020). Seeds were then planted in plastic pots (8 cm diameter × 7.5 cm high) containing the planting substrate (mixture of 0.5 Kg of peat compost and soil 1:5) (Mea Ltd., Nairobi, Kenya) with a volume of 0.5 L. The substrate was sterilized in an autoclave for 2 h at 121°C and allowed to cool for 72 h prior to planting. Five seeds were sowed per pot and maintained at room temperature (25 ± 2°C, 60% RH and 12:12 L:D photoperiod). Pots were transferred immediately after germination to the screen house (2.8 m length × 1.8 m width × 2.2 m height) at 25 ± 2°C, 55% RH, and 12:12 L:D photoperiod for 4–5 weeks. After germination, seedlings were thinned to two per pot and watered twice per day (morning and evening). No additional fertilizer was added to the planting substrate. Plants of 4–5 week-old were used for the various experiments.

To confirm endophytic colonization before the experiments, tomato plants were carefully uprooted from the pots 4–5 weeks post-inoculation and washed under running tap water to remove any soil attached to the plants. Seedlings (ca. 30 cm in height) were divided into three different sections (ca. 5 cm long): leaves, stems, and root using a sterile scalpel (Akutse et al., 2013; Agbessenou et al., 2020). Five sections each from the leaf, stem, and root of each plant were randomly selected and surface -sterilized as previously described for seeds. The different plant parts were then aseptically cut under a laminar flow hood into 1 × 1 cm pieces and placed four cm apart from each other on PDA plates supplemented with a 0.05% solution of antibiotic (streptomycin sulfate salt) (Akutse et al., 2013). Plates were incubated at 25 ± 1°C for 10 days, after which the presence of endophyte was determined. In addition to the last rinse water that was plated, plate imprinting was also conducted to assess the effectiveness of surface sterilization of plant materials (Inglis et al., 2012). The colonization of the different plant parts was recorded by counting the number of pieces of plant parts that showed the presence of inoculated fungal growth/mycelia according to Koch’s postulates (Petrini and Fisher, 1986). Only the presence of endophyte that was inoculated was scored from each incubated plate. Fungal isolate was identified morphologically using slides which were prepared from the mother plates. Treatments (non-inoculated and inoculated plants) were arranged in a randomized complete block design (RCBD) with four replicates. The success rate of fungal endophyte colonization (%) of tomato host plant parts was calculated as follows:


(1) $$\text{Colonization}\left(\%\right)=\frac{\begin{array}{c} \mathrm{Number}\,\mathrm{of}\,\mathrm{pieces}\\ \mathrm{exhibiting}\,\mathrm{fungal}\,\mathrm{outgrowth} \end{array}}{\mathrm{Total}\,\mathrm{number}\,\mathrm{of}\,\mathrm{pieces}\,\mathrm{plated}\,\mathrm{out}}\times 100$$

### Insects

A colony of *T. absoluta* was established from wild adults and larvae collected from infested tomato leaves and fruits in Mwea (0° 36′ 31.3′′ S 037° 22′ 29.7′′ E), Kenya in June 2019. The moths were kept in ventilated, sleeved Perspex cages (40 cm × 40 cm × 45 cm) and were fed *ad libitum* with 10% honey solution placed on the top side of each cage as food source (Agbessenou et al., 2020). *Tuta absoluta* were reared on non-inoculated tomato plants which were grown under screen house conditions at 25 ± 2°C, 65 ± 10% RH at *icipe*. Tomato nurseries were established by sowing seeds on a mixture of 5:1 soil: manure (i.e., 10 g of peat compost) in a seed raising plastic tray. Three weeks later, tomato seedlings were transplanted on a 5:1 soil: manure mixture (i.e., 0.5 Kg of peat compost) in plastic pots (8 cm diameter × 7.5 cm high) at a density of two plants per pot and watered as needed. Three weeks after transplanting, four potted non-inoculated tomato plants were placed in the rearing cages for oviposition. The plants were removed 24 h post-exposure and transferred to separate wooden cages (50 cm × 50 cm × 60 cm) ventilated with netting material at the sides and on the top until the eggs hatched. Leaves with larvae were removed from these plants, 3 days after hatching and placed into clean sterile plastic containers (21 cm long × 15 cm wide × 8 cm high) lined with paper towel to absorb excess moisture and fine netting infused lid for ventilation. The larvae were supplied daily with fresh tomato leaves (free from endophytes) as food until they pupated. The pupae were collected from the plastic containers using a fine camel hair-brush and placed inside a clean plastic container for adult emergence. The colony was rejuvenated every 3 months through infusion, with infested tomato leaves collected from the field to reduce inbreeding (Agbessenou et al., 2020; Akutse et al., 2020). Insects were maintained under a controlled rearing condition of 28 ± 2°C, 48% RH, and 12:12 L:D photoperiod in the laboratory at the Animal Rearing and Quarantine Unit (ARQU) of *icipe* for five generations prior to bioassays.

### Behavioral Responses of Females of *Tuta absoluta* to Inoculated Tomato Plant

#### Y-Tube Olfactometer Bioassays

The responses of females of *T. absoluta* to volatiles of non-inoculated, inoculated, non-inoculated infested, and inoculated infested tomato plants were tested in dual-choice assays using a vertically-oriented Y-tube olfactometer (3 cm internal diameter; 15 cm stem; two 10 cm side arms) (Sigma Scientific, Gainesville, FL, United States) connected with Teflon tubing to the test odor sources which were headspace of tomato plant volatiles. The control arm was an empty oven bag (25 cm × 380 cm, Baco & BacoFoil, Wrap- Film Systems Ltd., United Kingdom). Depending on the experiments, each arm of the olfactometer is connected to either the tomato plant volatiles or an empty oven bag with Teflon tubing. A vacuum pump (Cole-Parmer Instrument Co., Chicago, IL, United States) was used to generate the air which was filtered by an active charcoal filter and passed through each odor source tied in an oven baked bag at a constant flow rate of 150 mL min^–1^. Infested plants were obtained by infesting 4-week-old plant with 20 *T. absoluta* second instar larvae for 7 days. Potted plants were wrapped in aluminum foil to avoid contamination by volatiles from the pot and soil. To eliminate visual cues from the bioassay arena, the olfactometer was illuminated using 20 W red fluorescent tubes placed at a height of 0.5 m above the device (Sokame et al., 2019). Individual insects were released into the stem of the olfactometer and allowed 5 min to settle and another 5 min to make a choice. An insect was considered to have made a choice when it walked and reached the end of a given arm and remained there for at least 20 s. Insects which did not make a choice within 5 min were considered as non-respondent and were subsequently excluded from the statistical analysis. After every five tests, the odor sources were interchanged to eliminate positional bias. After every 10 replicates, a Y-tube and new test odor were used. Y-tubes were cleaned with Teepol odorless detergent (Sudi Chemical Industries Ltd, Nairobi, Kenya) rinsed with distilled water and dried in an oven overnight at 80°C before use. All experiments were conducted at 26 ± 1°C and 48–60% RH during the scotophase 1,800–2,000 h in sync with the behavior and activity of *T*. *absoluta* which has been shown to be active during this time (Proffit et al., 2011; Ataide et al., 2017). Responses of 2-day-old mated gravid females of *T. absoluta* toward the different odor sources are presented in Table 1. Forty insects were tested per odor combination and a total of 440 insects used in the whole experiment.

**TABLE 1:** Summary of odor sources tested in the Y-tube olfactometer assays.

Experiment	Odor sources
A	Air vs. air (control)
B	Air vs. non-inoculated plant
C	Air vs. inoculated plant
D	Air vs. non-inoculated infested plant
E	Air vs. inoculated infested plant
F	Non-inoculated plant vs. inoculated plant
G	Inoculated infested plant vs. inoculated infested plant
H	Inoculated plant vs. non-inoculated infested plants
I	Inoculated plant vs. inoculated infested plants
J	Non-inoculated plant vs. inoculated infested plants
K	Inoculated plant vs. non-inoculated infested plant

#### Collection of Volatiles

Headspace volatiles were trapped from 4-week-old tomato plants using a push-pull entrainment system (Analytical Research System, Gainesville, FL, United States) at night for 12 h onto a Super-Q adsorbent (30 mg, Analytical Research System, Gainesville, FL, United States) preconditioned using dichloromethane and dried under nitrogen by passing charcoal-purified air through tomato leaves tied in an oven baked bag at 350 mL/min. Volatiles were collected from non-inoculated, inoculated, non-inoculated infested, and inoculated infested tomato plants, as well as from an empty oven bag with no plant (control). This was repeated three times with two plants per replicate. Volatiles were eluted into a 2 mL sample vial using 150 μL of dichloromethane (Analytical grade, Sigma-Aldrich, St, Louis, MO, United States) and stored at −80°C until required for chemical analysis.

#### Analysis of Volatiles

One μL aliquot of each volatile extract was injected onto a gas chromatograph coupled mass spectrometer (GC-MS) in a splitless mode. The GC was equipped with a non-polar HP-5 MSI ultra-inert column (30 m × 0.25 mm i.d., 0.25 μm film thickness) (J&W, Folsom, CA, United States) with helium as the carrier gas at a flow rate of 1.2 mL min^–1^. The oven temperature was held at 35°C for 5 min, then programmed to increase at 10°C min^–1^ to 280°C and was maintained at this temperature for 10.5 min. The mass selective detector was maintained at ion source temperature of 230°C and a MS quadrapole temperature of 150°C. Spectra were recorded at 70 eV in the electron impact (EI) ionization mode. Fragment ions was analyzed over 40–550 *m/z* mass range in the full scan mode. Compounds were identified by comparing their mass spectra with those from the libraries Adams2, Chemecol and NIST11 search program (v. 2.0) and NIST Chemistry Webbook and retention indices using retention times of a mixture of n-alkanes (C_8_–C_31_). Where available, the identities of compounds were confirmed by co-injection with commercially available authentic standards. Quantification was achieved using external calibration curves made from 1,000 ng/μL stock solutions of the monoterpene β-pinene and the sesquiterpenes (*E*)-β-caryophyllene in a range of concentrations from 0.1 to 1,000 ng/μL. Concentration of compounds were computed by extrapolating the peak area of the unknown against those of the known concentration and expressed in ng/plant/h.

#### Chemicals

Authentic standards of (*E*)-2-hexenal, *p*-xylene, α-pinene, β-pinene, trans-isolimonene, β-myrcene, 2-carene, α-phellandrene, 3-carene, α-terpinene, *p*-cymene, β-phellandrene, (*Z*)-β-ocimene, (*E*)-β-ocimene, γ-terpinene, terpinolene, n-nonanal, allo-ocimene, methyl salicylate, β-elemene, (*Z*)-jasmone, (*E*)-β-caryophyllene, and α-humulene (>95% purity) were purchased from Sigma-Aldrich (St. Louis, MO, United States). Dichloromethane (99.9% purity) was purchased from Merck (Germany).

#### Bioassays With Synthetic Compounds

Of the volatiles identified from inoculated tomato plant: α-pinene, β-phellandrene, 2-carene, and (*E*)-β-caryophyllene were selected and tested based on results obtained from a similarity percentage (SIMPER) analysis. Methyl salicylate was also tested because it has previously been reported to elicit activity in antennae of females *T. absoluta* (Anastasaki et al., 2018). The behavioral responses of *T. absoluta* females were tested using a Y-tube olfactometer similar to the one used for initial behavioral bioassays. Three concentrations of each compound were tested in their natural release rate (ng/plant/h) from inoculated tomato plant, then by doubling and halving the amounts of each individual compound. Based on the results, three compounds (α-pinene, 2-carene, and β-phellandrene) were found attractive to females of *T. absoluta* and were tested in three-component blend at three concentrations against the control and methyl salicylate which was repellent to the females (2.17 ng/μL): (1.86 ng/μL α-pinene, 53.94 ng/μL 2-carene, 74.87 ng/μL β-phellandrene) (blend B1) which was subsequently doubled (blend B2) and diluted to one-half (blend B3) (Njuguna et al., 2018; Kihika et al., 2020). In another set of experiment, methyl salicylate (2.17 ng/μL) was tested against non-inoculated tomato plant. Thereafter, a four-component blend (B4) comprised of optimal attractant/repellent concentrations of the individual compound (1.86 ng/μL α-pinene, 53.94 ng/μL 2-carene, 74.87 ng/μL β-phellandrene, and 2.17 ng/μL methyl salicylate) was formulated and tested against the control. Blend B4 was subsequently doubled (blend B5) and diluted to one-half (blend B6) (Table 2). Each individual compound and blends were diluted in dichloromethane and a 50 μL aliquot of the test solution was applied on a filter paper (3 × 3 cm) (Whatman, United Kingdom) and tested against the control (i.e., filter paper loaded with 50 μL dichloromethane). The solvent was allowed to evaporate at room temperature for 30 s prior to the bioassays. Thereafter, the impregnated filter papers were placed at the edge of the olfactometer arms and renewed for every insect. Forty insects were tested per choice and a total of 1,000 insects at the end.

**TABLE 2:** Summary of blends tested in the Y-tube olfactometer assays.

Blend type	Blend composition
Blend B1	α-pinene (1.86) + 2-carene (53.94) + β-phellandrene (74.87)
Blend B2	α-pinene (3.73) + 2-carene (107.88) + β-phellandrene (149.74)
Blend B3	α-pinene (1) + 2-carene (26.97) + β-phellandrene (37.44)
Blend B4	α-pinene (1.86) + 2-carene (53.94) + β-phellandrene (74.87) + methyl salicylate (2.17)
Blend B5	α-pinene (3.73) + 2-carene (107.88) + β-phellandrene (149.74) + methyl salicylate (4.34)
Blend B6	α-pinene (1) + 2-carene (26.97) + β-phellandrene (37.44) + methyl salicylate (1.09)

*Numbers in parenthesis indicate concentration of each compound in ng/μL.*

### Herbivory Feeding Bioassay With (*Z*)-jasmone

Tomato leaflet bioassay using first instar *T. absoluta* larvae was conducted to assess the herbivore response. Fresh tomato leaflets were treated with aqueous (*Z*)-jasmone solutions at three different concentrations (1, 10, and 100 ng/μL) using a Potter Precision Laboratory Spray Tower (Burkard Manufacturing Co., Rickmansworth, United Kingdom) at constant air pressure of 10 PSI. Control leaflets were treated with sterile distilled water. First instar larvae (*n* = 10/treatment) were individually weighed and transferred to plastic Petri dishes (9 cm diameter) with fresh treated or untreated tomato leaflets. The petioles were wrapped in cotton cloth to maintain turgidity. The Petri dishes were sealed with Parafilm and incubated at 25 ± 2°C and 45 ± 1% RH. Every 3 days, leaflets were replaced to ensure fresh food for the larvae. Larval mortality was recorded daily for 7 days. The experiment consisted of 10 insects per treatment and was replicated four times.

### Statistical Analyses

All statistical analyses were performed using R statistical software, version 3.6.3 (R Core Team, 2019) and PAST, version 4.02. Females of *T. absoluta* preference for odors in the Y-tube olfactometer was assessed by comparing the recorded frequencies of choice of either of the olfactometer arms using a chi-square (χ^2^) test. Concentrations of volatile compounds between the four treatments including non-inoculated, inoculated, non-inoculated infested, and inoculated infested tomato plants were analyzed using the non-parametric Kruskal-Wallis test because the data were not normally distributed. Whenever there was a significant difference, a *post-hoc* Dunn’s test was performed for mean separation with Bonferroni’s adjustment. Principal components analysis (PCA) and non-metric multi-dimensional scaling (NMDS) was used to visualize the profile of identified headspace volatiles. The headspace chemical profiles from the four treatments were compared using one-way ANOSIM with the Bray–Curtis dissimilarity index. Similarity percentage (SIMPER) analysis was performed on peak areas of volatile compounds to determine the relative contribution of different compounds to the dissimilarity among volatiles of the different treatments. Cumulative mortality was corrected using Abbott’s formula (Abbott, 1925). Mortality data were analyzed using logistic regression in a GLM for a binomial distribution. Lethal time (LT_50_) was estimated by probit analysis using the package “ecotox” (Wheeler et al., 2006).

## Results

### Endophytic Colonization of Tomato Plant by *Trichoderma asperellum* M2RT4

**FIGURE 1:**
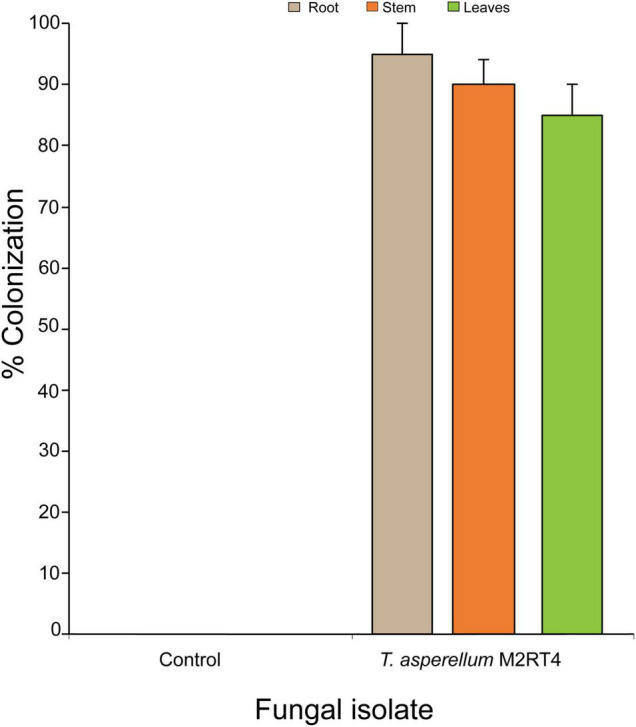
Endophytic colonization of tomato *Solanum lycopersicum* host plant parts by *Trichoderma asperellum* M2RT4 at 4–5 weeks post-inoculation. Bar chart represents means ± SE (standard error) at 95% CI (*P* < 0.05; *n* = 4).

*Trichoderma asperellum* M2RT4 successfully colonized 95, 90, and 85% of roots, stems, and leaves of tomato plant, respectively (Figure 1). Additionally, no fungal outgrowth was observed in the non-inoculated plant (Figure 1).

### Olfactory Response of Females of *Tuta absoluta* to Tomato Plant Volatiles

**FIGURE 2:**
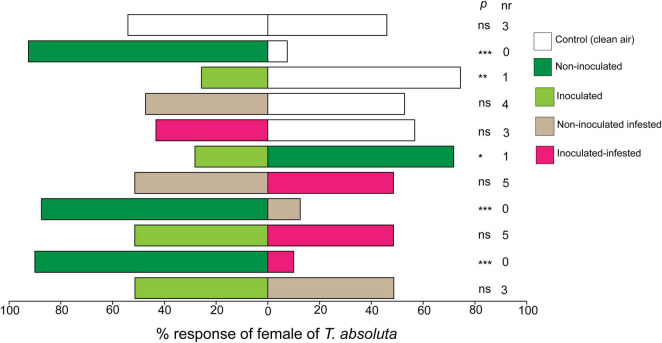
Responses (%) of females of *Tuta absoluta* to volatiles from non-inoculated, inoculated, non-inoculated infested, and inoculated infested tomato plants in a Y-tube olfactometer choice test. nr, number of non-respondent insects (i.e., no choice). *P* stands for statistical significance levels with ns, no significant difference (*P* > 0.05); *, **, ***, significant differences at *P* < 0.05; *P* < 0.01; and *P* < 0.001, respectively, from χ^2^ test at α = 0.05.

Females of *T. absoluta* were significantly attracted to volatiles from non-inoculated tomato plants compared to clean air (control) (χ^2^ = 27.22, df = 1, *P* < 0.0001) (Figure 2). In contrast, *T. absoluta* females significantly avoided inoculated tomato plant volatiles (χ^2^ = 8.30, df = 1, *P* < 0.001) (Figure 2). On the other hand, females of *T. absoluta* were not attracted to non-inoculated infested (χ^2^ = 0.02, df = 1, *P* = 0.86) or inoculated infested tomato plants (χ^2^ = 0.43, df = 1, *P* = 0.51) when compared to clean air (Figure 2). In paired assays, *T. asboluta* females were more attracted to the odor of non-inoculated tomato plant than to those of inoculated plants (χ^2^ = 6.56, df = 1, *P* < 0.01) (Figure 2). Similarly, *T. absoluta* females significantly preferred odors from non-inoculated plants compared to non-inoculated infested plants (χ^2^ = 21.02, df = 1, *P* < 0.001) and to inoculated plants (χ^2^ = 24.02, df = 1, *P* < 0.001) (Figure 2). However, *T. absoluta* females didn’t show any preference between non-inoculated infested and inoculated infested plants (χ^2^ = 0.78, df = 1, *P* = 0.18), neither between inoculated and inoculated infested plants (χ^2^ = 1.34, df = 1, *P* = 0.84), nor between inoculated and non-inoculated infested plants (χ^2^ = 2.34, df = 1, *P* = 0.49) (Figure 2).

### Analysis of Volatiles

**FIGURE 3:**
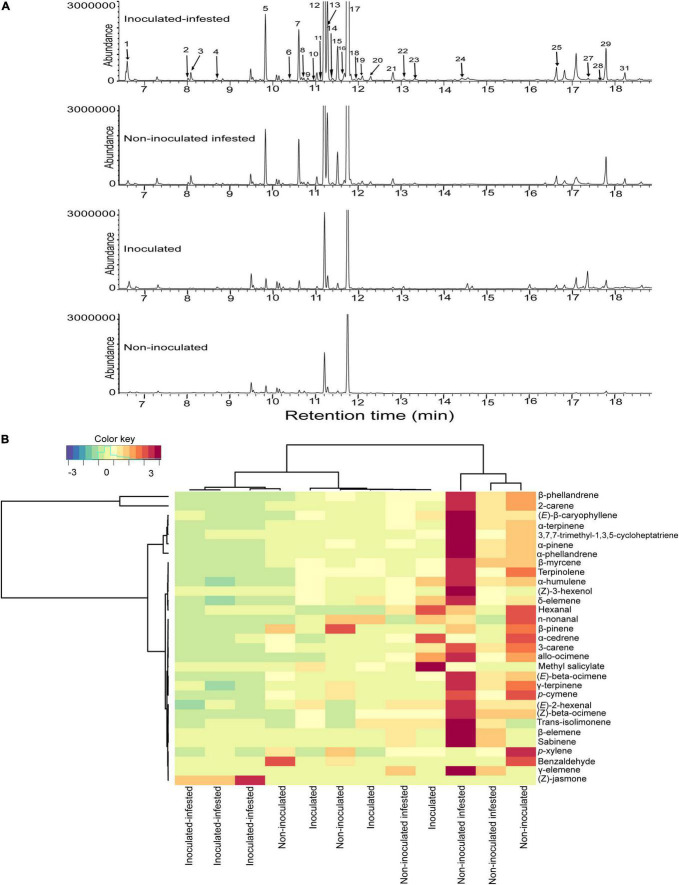
Variations in volatile organic compounds across non-inoculated, inoculated, non-inoculated infested, and inoculated infested tomato plants. **(A)** Representative total ion chromatogram of non-inoculated, inoculated, non-inoculated infested, and inoculated infested tomato plants. **(B)** Heatmap clustering showing the abundance (in decreasing color intensity) of the volatile compounds across replicates of the four treatments.

**FIGURE 4:**
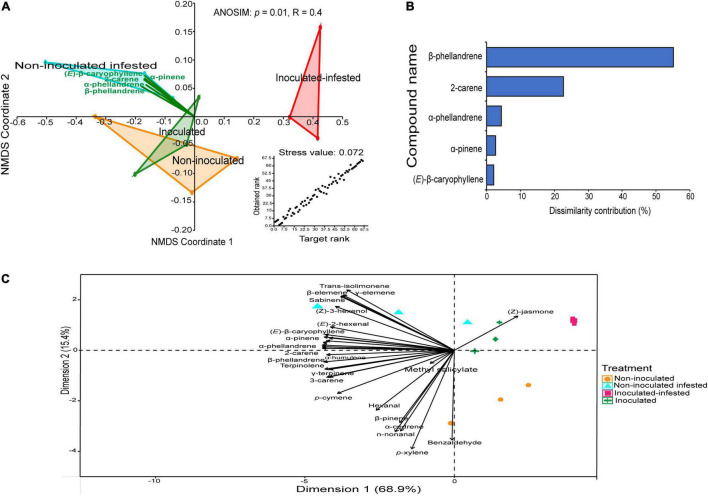
Variations in volatile organic compounds across the four treatments. **(A)** Non-metric multidimensional scaling analysis (NMDS) of the volatile pattern of non-inoculated, inoculated, non-inoculated infested, and inoculated infested tomato plants showing the variation in volatiles. **(B)** Similarity percentage analysis and the percent contribution of the predominant compounds for the dissimilarity between the four treatments. **(C)** Principal component analysis (PCA) of the volatile profiles produced from non-inoculated, inoculated, non-inoculated infested, and inoculated infested tomato plants.

A bouquet of 31 compounds representing eight chemical classes: monoterpenes (16), sesquiterpenes (6), aldehydes (4), alcohol (1), benzoid (1), homoterpene (1), ester (1), and ketone (1) were detected, identified and quantified in the volatile profiles of non-inoculated, inoculated, non-inoculated infested, and inoculated infested plants (Table 3 and Figure 3). Quantitative and qualitative differences in volatile emission were observed between non-inoculated, inoculated, non-inoculated –infested, and inoculated infested plants (Table 3 and Figures 3A,B). The monoterpenes 2-carene and β-phellandrene were the most abundant VOCs identified in the four treatments. The concentrations of α-pinene, 2-carene, α-phellandrene, and β-phellandrene were significantly higher in non-inoculated infested plants than in non-inoculated, inoculated and inoculated-infested plants. One compound (benzaldehyde) was only found in both non-inoculated and inoculated infested plants while three compounds (sabinene, δ- and γ-elemene) were only emitted by non-inoculated infested plants (Table 3). Additionally, methyl salicylate emission was 2-fold more abundant in inoculated plant than in non-inoculated infested and inoculated infested plants (Table 3). Further, (*Z*)-jasmone was only detected from inoculated infested tomato plants (Table 3). Compounds that did not differ significantly in the emission rate among the four treatments included hexanal, (*E*)-2-hexenal, *p*-xylene, β-myrcene, 3-carene, (*E*)-β-ocimene, γ-terpinene, terpinolene, n-nonanal, allo-ocimene, α-cedrene, (*E*)-β-caryophyllene, and α-humulene (Table 3). The NMDS clustered the four treatments successfully into four groups based on their VOC profiles (ANOSIM, *P* < 0.01) with five compounds [β-phellandrene (53.93%), 2-carene (19.91%), α-phellandrene (3.81%), (*E*)-β-caryophyllene (2.59%), and α-pinene (2%)] contributing the most to explaining the variation (Figures 4A,B). Furthermore, principal component analysis (PCA) of the VOCs showed that the first two components accounted for 68.9 (PC1) and 15.4% (PC2) of the total variation between the four treatments (Figure 4C). Dimension 1 was correlated with α-pinene, 2-carene, (*E*)-β-caryophyllene, α-phellandrene, and β-phellandrene while dimension 2 was correlated with benzaldehyde.

**TABLE 3:** Mean amount (ng/plant/h) of volatile compounds identified in the headspace of non-inoculated, inoculated, non-inoculated infested, and inoculated infested tomato plants (*n* = 3).

Peak no.	RT	RI_alk_^a^	RI_lit_^b^	Compound^c^	Chemical class	Non-inoculated plant	Inoculated plant	Non-inoculated infested plant	Inoculated infested plant	*P*-value^d^
1	6.64	807	801	Hexanal	Aldehyde	15.96 ± 6.91	15.22 ± 7.13	16.42 ± 2.16	8.56 ± 1.12	0.361
2	8.03	860	856	(*E*)-2-hexenal*	Aldehyde	7.46 ± 0.46	7.81 ± 0.41	9.47 ± 1.02	6.71 ± 0.33	0.098
3	8.11	863	860	(*Z*)-3-hexenol	Alcohol	7.67 ± 0.28 b	8.5 ± 0.34 b	17.22 ± 6.89 a	6.51 ± 0.08 b	**0.018**
4	8.37	873	865	*p*-xylene*	Benzoid	8.55 ± 0.69	6.52 ± 0.27	7.01 ± 0.06	6.44 ± 0.09	0.06
5	9.83	936	934	α-pinene*	Monoterpene	35.67 ± 10.3 ab	23.31 ± 3.11 ab	63.33 ± 27.42 a	12.74 ± 1.76 b	**0.033**
6	10.42	963	963	Benzaldehyde	Aldehyde	8.79 ± 0.62	nd	nd	6.51 ± 0.06	**0.014**
7	10.61	972	972	3,7,7-trimethyl-1,3,5-cycloheptatriene	Homoterpene	25.69 ± 9.6 ab	18.75 ± 3.07 ab	52.7 ± 26.24 a	11.56 ± 1.69 b	**0.043**
8	10.67	975	974	Sabinene	Monoterperne	nd	nd	9.76 ± 1.54	nd	–
9	10.73	978	978	β-pinene*	Monoterperne	14.69 ± 1.48 a	7.96 ± 0.18 ab	9.46 ± 1.34 ab	6.77 ± 0.1 b	**0.027**
10	10.83	983	983	trans-isolimonene*	Monoterperne	nd	8.4 ± 0.29 ab	10.46 ± 1.76 b	6.78 ± 0.08 ab	**0.017**
11	11.037	992	992	β-myrcene*	Monoterperne	12.12 ± 2.54	12.92 ± 0.45	18.75 ± 4.72	7.15 ± 0.13	0.057
12	11.21	1001	1001	2-carene*	Monoterperne	253.84 ± 123.79 ab	168.57 ± 10.2 ab	480.06 ± 204.04 a	58.38 ± 10.27 b	**0.043**
13	11.28	1005	1005	α-phellandrene*	Monoterperne	52.67 ± 23.81 ab	36.81 ± 1.72 ab	97.96 ± 41.45 a	15.87 ± 1.49 b	**0.043**
14	11.41	1011	1011	3-carene*	Monoterperne	9.59 ± 1.51	8.65 ± 0.63	10.59 ± 1.75	6.99 ± 0.12	0.086
15	11.52	1018	1018	α-terpinene*	Monoterperne	26.84 ± 10.3 ab	19.96 ± 0.8 ab	48.23 ± 19.04 a	9.96 ± 0.74 b	**0.043**
16	11.67	1026	1026	*p*-cymene*	Monoterperne	13.52 ± .92 a	8.93 ± 0.18 ab	12.7 ± 3.31 ab	7.29 ± 0.09 b	**0.038**
17	11.75	1031	1032	β-phellandrene*	Monoterperne	698.16 ± 306.67 ab	467.93 ± 20.98 ab	1071.57 ± 385.23 a	158.65 ± 19.51 b	**0.044**
18	11.90	1039	1039	(*Z*)-β-ocimene*	Monoterperne	7.17 ± 0.88 ab	7.35 ± 7.35 ab	9.17 ± 0.93 a	nd	**0.041**
19	12.09	1050	1050	(*E*)-β-ocimene*	Monoterperne	10.71 ± 1.4	9.57 ± 0.41	13.82 ± 3.0	7.6 ± 0.24	0.063
20	12.29	1060	1060	γ-terpinene*	Monoterperne	11.87 ± 1.87	9 ± 0.22	12.7 ± 2.58	7.49 ± 0.52	0.061
21	12.80	1089	1090	Terpinolene*	Monoterperne	13.91 ± 4.26	11.92 ± 0.38	17.17 ± 5.59	6.75 ± 0.06	0.086
22	13.06	1104	1102	n-nonanal*	Aldehyde	12.78 ± 3.15	12.05 ± 1.9	8.31 ± 1.48	6.66 ± 0.08	0.055
23	13.33	1121	1121	Allo-ocimene*	Monoterperne	8.85 ± 1.64	8.85 ± 1.64	10.93 ± 2.27	6.39 ± 0.02	0.064
24	14.55	1198	1199	Methyl salicylate*	ar-Ester	nd	13.54 ± 4.83 b	7.66 ± 0.83 a	6.4 ± 0.04 a	**0.018**
25	16.63	1344	1342	δ-elemene	Sesquiterpene	12.27 ± 2.77	17.35 ± 1.36	20.83 ± 6.2	8.16 ± 0.7	0.082
26	17.37	1398	13.97	β-elemene*	Sesquiterpene	nd	nd	9.96 ± 1.5	nd	–
27	17.49	1407	1403	(*Z*)-jasmone*	Ketone	nd	nd	nd	6.47 ± 0.06	–
28	17.72	1426	1424	α-cedrene	Sesquiterpene	10.3 ± 2.08	9.62 ± 2.92	8.19 ± 0.32	6.62 ± 0.28	0.218
29	17.79	1432	1430	(*E*)-β-caryophyllene*	Sesquiterpene	23.75 ± 7.6	30.29 ± 4.38	47.54 ± 18	13.94 ± 2.35	0.121
30	17.91	1441	1441	γ-elemene	Sesquiterpene	nd	nd	7.53 ± 0.46	nd	–
31	18.23	1467	1465	α-humulene*	Sesquiterpene	11.63 ± 2.56	13.26 ± 2.19	16.69 ± 4.07	7.93 ± 0.5	0.075

*^a^Retention index relative to C8–C23 n-alkanes of an Inert Cap 5MS/NP capillary column.*

*^b^Retention index obtained from the literature (Khan et al., 2012).*

^c^Identification of compounds based on the retention time (RT) and comparison of mass spectra with published mass spectral library data from NIST11 and Wiley9. *indicates compounds confirmed with authentic standards.

*^d^P-value of the non-parametric Kruskal Wallis test for comparison of volatile compounds between non-inoculated, inoculated, non-inoculated infested, and inoculated infested tomato plants. Significant values are highlighted in bold, where means (±SE) followed by the same letters within rows are not significantly different.*

*nd, not detected.*

### Behavioral Response to Synthetic Compounds

**FIGURE 5:**
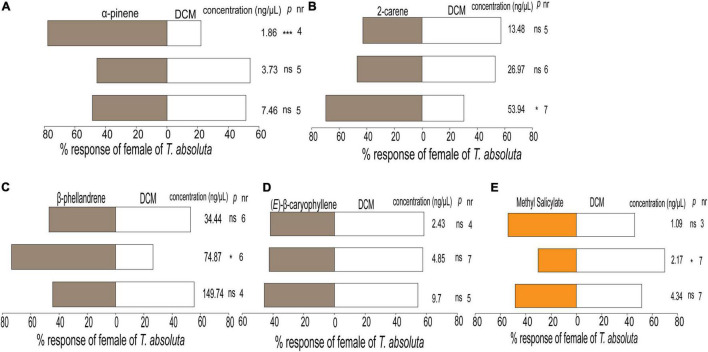
Behavioral responses of females of *Tuta absoluta* to odor components tested at three concentrations **(A)** α-pinene, **(B)** 2-carene, **(C)** β-phellandrene, **(D)** (*E*)-β-caryophyllene, and **(E)** methyl salicylate, all against DCM, dichloromethane (control solvent) in a Y-tube olfactometer. Each pair of evaluation used a total of *n* = 40 females of *Tuta absoluta* for each compound concentration released singly per choice. nr, number of non-respondent insects (i.e., no choice). *P* stands for statistical significance levels with ns, no significant difference (*P* > 0.05); *, ***, significant differences at *P* < 0.05 and *P* < 0.001, respectively, from χ^2^ test at α = 0.05.

Females of *T. absoluta* responded differently to the various concentrations of synthetic compounds tested individually compared to the control (Figure 5). The females significantly preferred α-pinene at 1.86 ng/μL (χ^2^ = 10.02, df = 1, *P* < 0.001, Figure 5A), 2-carene at 53.94 ng/μL (χ^2^ = 4.36, df = 1, *P* = 0.03, Figure 5B), and β-phellandrene at 74.87 ng/μL (χ^2^ = 6.61, df = 1, *P* < 0.01, Figure 5C) compared to the control. In contrast, (*E*)-β-caryophyllene was not attractive to the females at any of the tested concentrations (Figure 5D) while methyl salicylate repelled them at a concentration of 2.17 ng/μL (χ^2^ = 4.36, df = 1, *P* = 0.03) (Figure 5E). Hence this concentration was used in the subsequent experiments.

**FIGURE 6:**
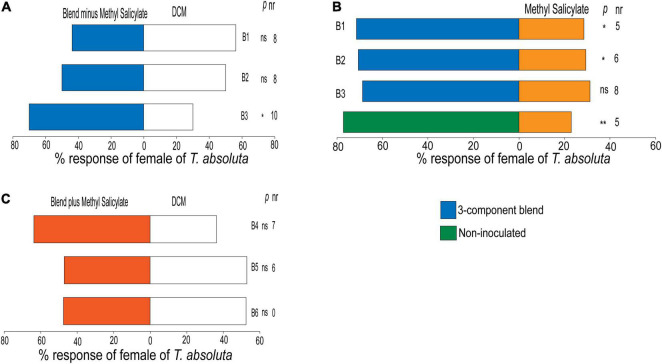
Behavioral responses of females of *Tuta absoluta* to **(A)** the three-component blend consisting of the most attractive compounds, α-pinene, 2-carene, β-phellandrene, all against DCM, dichloromethane (control solvent). **(B)** % responses of females of *T. absoluta* to three-component blend and non-inoculated tomato plant all against methyl salicylate (positive control). **(C)** % responses of females of *T. absoluta* to the four-component blend containing α-pinene, 2-carene, β-phellandrene, and methyl salicylate in a Y-tube olfactometer. nr, number of non-respondent insects (i.e., no choice). *P* stands for statistical significance levels with ns, no significant difference (*P* > 0.05); *, **, significant differences at *P* < 0.05; and *P* < 0.01, respectively, from χ^2^ test at α = 0.05.

Females of *T. absoluta* did not show any preference to the blend of the three attractants (α-pinene, 2-carene, and β-phellandrene) mixed at their attractive concentrations (blend B1) (χ^2^ = 0.28, df = 1, *P* = 0.59) or when the concentrations were doubled (blend B2) (χ^2^ = 0.33, df = 1, *P* = 0.87) compared to the control (Figure 6A). However, the blend B3 composed of one-half of the blend B1 concentration was relatively attractive to the females (χ^2^ = 4.03, df = 1, *P* = 0.04) (Figure 6A). Interestingly, females of *T. absoluta* preferred the blend of the three attractants (α-pinene, 2-carene, and β-phellandrene) mixed at their attractive concentrations (blend B1) (χ^2^ = 5.60, df = 1, *P* < 0.01) or when doubled (blend B2) (χ^2^ = 4.97, df = 1, *P* = 0.02) compared to methyl salicylate (Figure 6B). Surprisingly, females of *T. absoluta* were not attracted to the blend B3 composed of one-half of the blend B1 concentration compared to methyl salicylate (χ^2^ = 3.78, df = 1, *P* = 0.06) (Figure 6B). Also, *T. absoluta* females preferred volatiles of non-inoculated tomato plant (χ^2^ = 9.25, df = 1, *P* < 0.01) compared to methyl salicylate (Figure 6B). In addition, females of *T. absoluta* did not show any preference for the four-component blend (B4) comprised of optimal attractant/repellent concentrations of the individual compound (χ^2^ = 1.93, df = 1, *P* = 0.16), or when the concentrations were doubled (blend B5) (χ^2^ = 0.03, df = 1, *P* = 0.86) or halved (blend B6) (χ^2^ = 0.03, df = 1, *P* = 0.87) compared to the control (Figure 6C).

### Herbivory Feeding Bioassay With (*Z*)-jasmone

**FIGURE 7:**
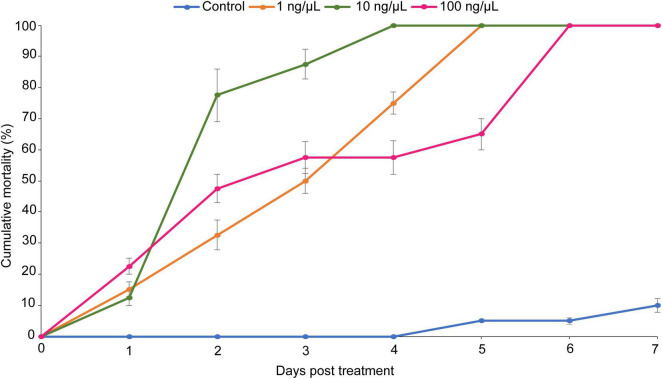
Cumulative larval mortality of *Tuta absoluta* induced by three concentrations (1, 10, and 100 ng/μL) of (*Z*)-jasmone.

Larval mortality varied significantly among the three concentrations (χ^2^ = 40.58, df = 3, *P* < 0.001) 7 days post-treatment (Figure 7). The concentration (10 ng/μL) had the highest (83%) larval mortality rate followed by the concentration at 1 ng/μL (67.5%) and at 100 ng/μL (64.24%) (Figure 7). Additionally, the concentration at 10 ng/μL had the shortest LT_50_ (1.73 days) (Table 4). Overall, (*Z*)-jasmone-treated tomato leaflets significantly reduced *T. absoluta* leafmining activity as compared to the control (Figure 7).

**TABLE 4:** LT_50_ values 7 days post-treatment of *Tuta absoluta* larvae exposed to different concentrations of (*Z*)-jasmone.

(*Z*)-jasmone concentration (ng/μL)	LT_50_ (days)^a^
1	2.72 (2.49–2.94)
10	1.73 (1.53–1.91)
100	2.79 (1.95–3.42)

*^a^Values in the bracket represent 95% Fiducial Limits (FL).*

## Discussion

Our results indicate that *in planta* colonization of tomato plant tissues by *T. asperellum* M2RT4 modifies the chemical response of the host plant affecting the abilities of *T. absoluta* females to locate their host. Specifically, we demonstrated that the endophytic fungus *T. asperellum* quantitatively and qualitatively alters tomato leaf volatiles composition by enhancing both the SA and JA plant defense pathways.

In the Y-tube olfactometer assays, we observed that females of *T. absoluta* avoided inoculated plants. This indicates that attraction of ovipositing females likely depends on the perception of *bona fide* semiochemicals, therefore orienting their movement toward odors released by suitable host plants. Previously, Ataide et al. (2017) reported that healthy tomato plant volatiles play an important role in modifying both flight and oviposition behavior of mated *T*. *absoluta* females. Clearly, plant volatiles of inoculated tomato plants are far less attractive to ovipositing females, as reflected in reduced oviposition on the inoculated plants (Agbessenou et al., 2020). Additionally, the preference of *T. absoluta* females toward non-inoculated plants in dual-choice assay suggests that they might perceive odors from inoculated plants which could signal unsuitable hosts leading to their avoidance and this is considered as a crucial step for the reproductive success of the pest. Our results also showed that *T*. *absoluta* females were not attracted neither to non-inoculated infested nor to inoculated infested tomato plants. This could be explained by the fact that *T*. *absoluta* females perceive the infested plant through chemical cues as poor sources for the development of their offspring. This is in agreement with De Moraes et al. (2001) who reported that *Heliothis virescens* (Fabricius) (Lepidoptera: Noctuidae) female moths exploit volatile signals from infested plants to avoid oviposition on previously damaged plants.

Our findings reveal the emission of a bouquet of VOCs largely dominated by monoterpenes and sesquiterpenes. Terpenes are known to play an important ecological role in plant-insect interactions, including serving as attractants or repellents to herbivores, parasitoids, and predators (von Mérey et al., 2013; Helms et al., 2017). For example, α-pinene, α-phellandrene, and β-phellandrene are shown to be attractants to the predator *Nesidiocoris tenuis* (Hemiptera: Miridae) (Ayelo et al., 2021b). Notably, we found an increase in the emission of terpenoids by infested plants upon herbivory by *T. absoluta*, with 2-carene and β-phellandrene as the most abundant VOCs similar to findings by Silva et al. (2018). Earlier on, Turlings et al. (1995) also reported an increase in the emission of terpenoids (α-pinene and β-phellandrene) in cotton and corn plants upon herbivory by the beet armyworm, *Spodoptera exigua* (Lepidoptera: Noctuidae). Turlings et al. (1995) speculated that the terpenoids are stored in the glands located in the leaves of cotton, the host plant which may serve as toxins to directly discourage herbivores from feeding on the leaves. Similarly, Gouinguené et al. (2003) reported an increase in terpenoid emissions in *Zea mays* which was elicited by oral secretions of *Spodoptera littoralis* (Lepidoptera: Noctuidae). Interestingly, we found that the quantitative variation in the emission of volatiles was only evident in the monoterpenes 2-carene, α-pinene, α-phellandrene, and β-phellandrene, but not in sesquiterpenes such as δ-elemene, α-cedrene, (*E*)-β-caryophyllene, and α-humulene. Similar findings on increase in the production of monoterpenes were also reported in below ground interactions between the tomato root-knot nematode *Meloidogyne javanica* and tomato plants (Kihika et al., 2020). It can therefore be speculated that increases in the release of monoterpenes by plants is triggered by low infestations which could serve as priming signals for activating plant defense systems/mechanisms. When present in tomato plants, *T. asperellum* alter the composition of volatile emissions, thereby inducing a quantitative difference between non-inoculated and inoculated plants. Specifically, we found that inoculated tomato plants elicited a reduction in the emission of several compounds including α-pinene, β-pinene, 2-carene, α-phellandrene, and β-phellandrene compared to non-inoculated plant. This is in contrast with Shrivastava et al. (2015) who reported an increase in the concentration of the monoterpenes (2-carene and sabinene) in *Beauveria bassiana*-inoculated tomato plants compared to the control. Conversely, we observed an increase even though non-significant in the emission of (*E*)-β-caryophyllene in inoculated plants compared to non-inoculated plants. This change in volatile profile was previously reported by Battaglia et al. (2013) who also observed an enhanced release of (*E*)-β-caryophyllene by *Trichoderma longibrachiatum* MK1-colonized tomato plants compared to the control. Interestingly, we found a significant reduction in the emission of most monoterpenes (α-pinene, 2-carene, α-phellandrene, α-terpinene, and β-phellandrene) by inoculated-infested plants compared to non-inoculated infested treatment.

The colonization of tomato plant by *T. asperellum* M2RT4 induces the release of trans-isolimonene and methyl salicylate which could probably explain the avoidance of inoculated plants by *T. absoluta* females. This is quite interesting as microbial infection is known to provide long term resistance to herbivore/pathogen attacks through the activation of the SA pathway (Shoresh et al., 2010). Although *T. asperellum* M2RT4 could have an intrinsic ability to colonize tomato host plant, its presence does not cause any harmful/negative effects to its host (Paradza et al., 2021). Therefore, its colonization of tomato triggered the defense mechanism of the plant leading to the emission of methyl salicylate. Conversely, previous studies have reported that the presence of endophytic fungi within host plants often leads to the inactivation of the plant salicylic acid pathway (Bastías et al., 2018). It is worth noting that one essential benefit this endophyte provides is that of a source of bioactive alkaloids and nitrogen-based compounds that protect host plants against herbivores (Bastías et al., 2018; Poveda, 2021b). The release of methyl salicylate by inoculated tomato plant indicates that the metabolic machinery of the plant is prepared for subsequent defense upon herbivory. Because of the high energy costs and nutrient requirements associated with the production and maintenance of defensive metabolites, the presence of endophytes helps the plant for protection against the pest without spending energy for self-defense or have energy to allocate to other functions (Thaler, 1999).

In this study, we observed that methyl salicylate is also released from infested plants supporting the suggestion that this compound could function as a signal associated with damage or induced defense (Hardie et al., 1994). Indeed, plants exhibit self-defense strategies against herbivores either directly, through negative effects on herbivore performance, or indirectly, by recruiting natural enemies of herbivores through the release of HIPVs (Moujahed et al., 2014). Previously, Silva et al. (2018) reported the emission of methyl salicylic upon *T. absoluta* herbivory. Salicylic acid is a stress-related hormone that has been reported to play an important role in the orchestration of plant defenses against herbivores (Shi et al., 2016). Interestingly, we observed that the level of methyl salicylate is higher in inoculated plants than in non-inoculated infested tomato plants. This protection mediated by methyl salicylate is a promising control strategy as the plants predominantly exhibit sessile-life style which exposes their vulnerability to biotic and abiotic stressors. Therefore, induction of resistance at the early stage (seed stage) may enhance the protection of tomato plant and suppress the population buildup of multivoltine pests like *T. absoluta* (Thaler et al., 2012; Strapasson et al., 2014).

We observed an emission of the key defense phytohormone (*Z*)-jasmone from inoculated infested plant. In contrast, Caparros Megido et al. (2014) and De Backer et al. (2015) reported the emission of (*Z*)-jasmone by non-inoculated infested tomato plants which we didn’t observe in this study. This could be attributed to its suppression through elicitors present in the oral regurgitant or saliva of *T. absoluta* immature stages. Several authors have previously reported the chemical response of tomato plant to *T. absoluta* herbivory and the signaling pathways involved (De Backer et al., 2017; Silva et al., 2018; Ayelo et al., 2021b). Besides, we observed a significant reduction in the abundance of monoterpenes as well as in the quantity of methyl salicylate in inoculated infested tomato plants compared to other treatments. This could be attributed to the activation of (*Z*)-jasmone signaling-pathway where tomato host plant responded to herbivore damage by producing (*Z*)-jasmone while reducing the quantity of methyl salicylate. Consistently, JA and SA have been reported as the dominant plant-signaling compounds that trigger induced resistance against herbivorous insects which most of the time render plants less susceptible to subsequent attack by pests (Cui et al., 2012). This supports results of our olfactometer bioassays where females of *T. absoluta* didn’t show any preference for infested plants. This confirms the observation that jasmonate synthesis is triggered by feeding damage from chewing herbivores (Cooper and Goggin, 2005).

The results of the herbivory feeding bioassay showed that (*Z*)-jasmone-treated tomato leaflets significantly reduced *T. absoluta* leafmining activity as compared to the control. Previously, Strapasson et al. (2014) reported that treatment of tomato seeds with methyl jasmonate enhances plant resistance at the seed stage which subsequently result in the reduction of the performance of *T. absoluta*. Apart from *T. absoluta* larvae, previous studies have shown the efficacy of (*Z*)-jasmone against immature stages of other insect pests. For instance, Sobhy et al. (2020) reported that priming potato plant with (*Z*)-jasmone negatively impacts the performance traits of aphids *Macrosiphum euphorbiae* (Thomas) (Hemiptera: Aphididae). Similarly, Black et al. (2003) reported the inhibitory effect of JA and this can be used as a vaccine against the leafminers (Diptera: Agromyzidae). The central role played by (*Z*)-jasmone in systemic feeding damage signaling in tomato plants underlines the relevance of treating tomato seeds with fungal inoculum so as to sustainably manage *T*. *absoluta*. Induction of (*Z*)-jasmone is known to attract parasitoids and predators of caterpillars (Turlings et al., 1990). However, these two defensive pathways (SA and JA) are not independent, and there could be antagonistic cross-talk between SA and JA pathways (Filgueiras et al., 2016). Therefore, it would be interesting to unravel the molecular mechanism underlying such interactions between endophyte and tomato plants against *T. absoluta* leading to activation of both SA and JA defense pathways and the production of methyl salicylate and (*Z*)-jasmone.

The response of an insect to a given compound is known to occur in dose-dependent manner (Kihika et al., 2017; Njuguna et al., 2018). In this study, we showed that females of *T. absoluta* responded differently to the concentrations of the individual compounds and the blend with and without methyl salicylate against the control. Individually, α-pinene, 2-carene, and β-phellandrene were attractive to females of *T. absoluta* at 1.86, 53.94, and 149.74 ng/μL, respectively, while (*E*)-β-caryophyllene did not elicit attraction of female moths at the three different concentrations tested. Interestingly, methyl salicylate was the only compound that elicited repellence behavior to *T. absoluta*. In addition, methyl salicylate’s significance in *T. absoluta* host location was evidenced when it was removed from the 3-component blend (α-pinene, 2-carene, and β-phellandrene) which resulted in the attraction of the moths. The repellent effect of methyl salicylate has previously been reported in aphids and the glasshouse whitefly (Xu et al., 2018; Conboy et al., 2020). Methyl salicylate has also being shown to induce defenses against *T. absoluta* (Pérez-Hedo et al., 2021) and serve as an oviposition attractant for the fall armyworm *Spodoptera frugiperda* (Lepidoptera: Noctuidae) (Yactayo-Chang et al., 2021).

In conclusion, our findings have shed light on the role of the endophyte *T. asperellum* M2RT4 in the activation of plant defense mechanisms in tomato plant through both the SA and JA pathways. Based on our results, we demonstrated that *T. asperellum* M2RT4 primes tomato plant defense in two ways. On the one hand, we found that colonization of tomato plant by *T. asperellum* M2RT4 induces the systemic release of methyl salicylate which has a repellent effect on the host location behavior of *T. absoluta* females. On the other hand, we also showed that *T. asperellum* M2RT4 triggered the release of (*Z*)-jasmone in inoculated infested tomato plants reducing the performance of the larval activity of the pest. This study has therefore laid the groundwork for future studies aimed at elucidating the underlying molecular mechanism that mediate endophyte-induced plant resistance against *T. absoluta*.

## Data Availability Statement

The original contributions presented in the study are included in the article/supplementary material, further inquiries can be directed to the corresponding author.

## Author Contributions

KA, AY, and FK supervised the experiment. AA performed the experiment, analyzed the data, and drafted the manuscript. KA and FK secured funding for the experiments. All authors conceived and designed the experiment, reviewed and edited the manuscript, read, and agreed to the published version of the manuscript.

## Author Disclaimer

The views expressed herein do not necessarily reflect the official opinion of the donors.

## Conflict of Interest

The authors declare that the research was conducted in the absence of any commercial or financial relationships that could be construed as a potential conflict of interest.

## Publisher’s Note

All claims expressed in this article are solely those of the authors and do not necessarily represent those of their affiliated organizations, or those of the publisher, the editors and the reviewers. Any product that may be evaluated in this article, or claim that may be made by its manufacturer, is not guaranteed or endorsed by the publisher.
